# Verschlussazoospermie bei unilateraler zystischer Alteration der Samenblase und kontralateraler Samenblasenagenesie – ein Fallbericht

**DOI:** 10.1007/s00120-025-02578-6

**Published:** 2025-04-14

**Authors:** Clarissa Wittler, D. Engeler, J. Rührup

**Affiliations:** Klinik für Urologie, Kantonsspital St. Gallen, Institut für Medizin, Universität St. Gallen, Rorschacher Straße 95, 9007 St. Gallen, Schweiz

**Keywords:** Genetische Ursachen der Azoospermie, Zystische Samenblase, Male infertility, Vas deferens, Spermatogenese, Genetic causes for azoospermia, Cystic seminal vesicle, Male infertility, Vas deferens, Spermatogenesis

## Abstract

Ein 29-jähriger Patient mit seit 2 Jahren bestehender primärer Infertilität stellte sich zur weiterführenden Abklärung vor. Wiederholte Spermiogramme zeigten eine Azoospermie bei saurem pH-Wert. Sonographisch zeigte sich ein unauffälliges Hodenparenchym bei normalem Hodenvolumen beidseits, die Samenstränge waren jedoch weder palpatorisch noch sonographisch sicher abgrenzbar. Im MRT zeigte sich ein zystisch erweitertes linkes Vas deferens, eine erweiterte linke Samenblase sowie ein nicht darstellbares rechtes Vas deferens. Eine testikuläre Spermienextraktion (TESE) zeigte eine regelhafte Spermiogenese mit einem Johnson-Score von 9.

## Abstract (en)

A 29-year-old man with primary infertility for 2 years presented for further evaluation. Repeated spermiograms showed azoospermia and an acidic pH. Sonographically, both testes showed normal size and parenchyma, but the vas deferens was bilaterally not palpable. Magnetic resonance imaging showed a cystically dilated left vas deferens and a dilated left seminal vesicle. The right vas deferens could not be visualized. Testicular sperm extraction (TESE) was successful and showed only slight impairment of the spermatogenesis with a Johnson score of 9.

## Anamnese

Ein 29-jähriger Patient stellte sich mit einem seit 2 Jahren bestehenden unerfüllten Kinderwunsch und primärer Infertilität in der Sprechstunde vor. Die Partnerin habe sich bereits einer unauffälligen gynäkologischen Untersuchung unterzogen.

Es bestand eine unauffällige Anamnese für Infektionen, Traumata oder Operationen im Urogenitalbereich bei Status nach Zirkumzision in der Kindheit. Es lagen seitens des Patienten keine Nebenerkrankungen vor und es wurden keine Medikamente eingenommen. Die Familienanamnese war ebenfalls unauffällig.

## Befund

Im körperlichen Untersuchungsbefund ergab sich ein regelrechter Ernährungs- sowie Allgemeinzustand ohne klinische Anzeichen eines Hypogonadismus, kein pathologisch auffallendes Behaarungsmuster oder Anzeichen einer Gynäkomastie. Darüber hinaus zeigte sich ein unauffälliger palpatorischer sowie sonographischer Befund beider Hoden. Die Samenleiter konnten jedoch nicht eindeutig palpiert werden. Bereits im Vorfeld der Erstkonsultation in unserer Klinik wurden zwei Spermiogramme im Abstand von 8 Wochen durchgeführt, diese zeigten eine Azoospermie bei Ejakulatvolumen im unteren Normbereich. Bei zudem erniedrigtem pH von jeweils 6,3 wurde der Verdacht auf das Vorliegen einer Verschlussazoospermie geäußert.

**Abb. 1:**
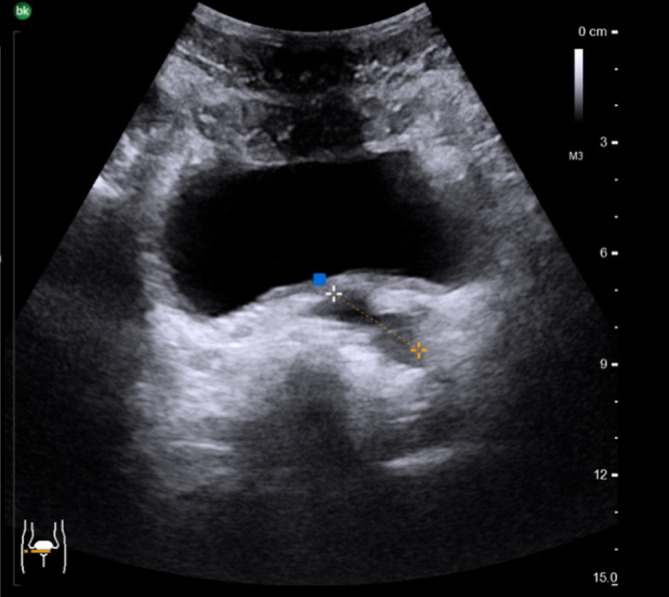
Sonographiebefund von transvesikal mit prominenter linker Samenblase dorsal der Harnblase

Sonographisch ließ sich eine prominente linke Samenblase im transvesikalen Ultraschall darstellen, sodass eine Bildgebung des Beckens mittels MRT durchgeführt wurde (Abb. 1 und 2). Hier zeigte sich ein zystisch dilatiertes Vas deferens sowie eine deutlich dilatierte Samenblase links bei nicht darstellbarem Vas deferens und Samenblasenagenesie rechts. Zum Ausschluss eines Zinner-Syndroms erfolgte neben einer Sonographie des oberen Harntrakts auch die weitere Diagnostik mittels MRT Abdomen. Bei regelrechtem Befund beider Nieren konnte ein Zinner-Syndrom ausgeschlossen werden.

Zur Vervollständigung der Diagnostik wurde eine genetische Analyse inklusive CFTR („cystic fibrosis transmemrane conductance regulator“)-Testung hinsichtlich Vorliegens einer zystischen Fibrose durchgeführt. Auch hier ergab sich kein pathologischer Befund.

Ein Hormonlabor ergab keinen Hinweis für eine Störung der Hormonachsen.

**Abb. 2:**
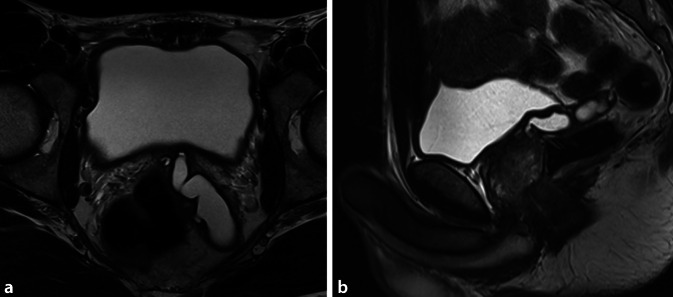
MRT Becken im Transversal- (**a**) und Sagittalschnitt (**b**) mit Anschnitt der zystisch erweiterten linken Samenblase

## Diagnose

Es lag eine Verschlussazoospermie bei Obstruktion durch die in der MRT nachgewiesene Samenblasenagenesie rechts und einen Samenblasenhydrops links vor.

## Therapie und Verlauf

Aufgrund des bestehenden Kinderwunsches konnte bei dem Patienten nach vorgängiger Aufklärung eine testikuläre Spermienextraktion (TESE) durchgeführt werden. Erfreulicherweise ergab sich hierbei histologisch ein reguläres, unauffälliges Hodenbiopsat mit histoanatomisch unauffälligen Hodenkanälchen und regelhafter Spermiogenese mit fokal minimaler Desorganisation im Sinne eines Johnson-Scores von 9. Es konnte ausreichend Material für eine im Verlauf geplante intrazytoplasmatische Spermieninjektion (ICSI) entnommen werden.

## Diskussion

Ein unerfüllter Kinderwunsch betrifft bis zu 10–15 % der Paare, die gezielte Diagnostik ist vor weiterer Therapieplanung essentiell. Bei nicht eindeutig tastbarem und radiologisch nicht darstellbarem Ductus deferens sollte vor weiterer Therapieplanung wie in unserem Patientenfall nach Ursachen für eine mögliche Samenblasen- und Dukutsagenesie gesucht werden.

Als mögliche Ursache einer Verschlussazoospermie ist neben Infektionen, Traumata und Operationen, die zystische Fibrose als Ursache zu bedenken [6]. In etwa 3 % der Fälle einer Azoospermie lässt sich in der weiteren Abklärung eine Mutation im CFTR nachweisen, welche zu einer Verschlussazoospermie durch eine kongenitale bilaterale Aplasie der Samenleiter (CBAVD) führt [7]. Diese mildeste Variante einer zystischen Fibrose manifestiert sich häufig erst durch Infertilität und einen unerfüllten Kinderwunsch. Vor weiterer Kinderwunschbehandlung sollte im Falle einer CBAVD auch eine genetische Beratung der Partnerin erfolgen, da die Mutation des CFTR autosomal-rezessiv vererbt wird. In unserem Fall konnte eine zystische Fibrose durch genetische Testung ausgeschlossen werden. Aufgrund des bildmorphologisch auffälligen, zystisch dilatierten Vas deferens und der dilatierten linken Samenblase sahen wir auch die Indikation zum Ausschluss eines Zinner-Syndroms als gegeben an. Beim Zinner-Syndrom handelt es sich um einen kongenitalen Fehlbildungskomplex des männlichen Urogenitalsystems, bestehend aus der Trias einer unilateralen Zyste der ipsilateralen Samenblase, Obstruktion des Vas deferens und Agenesie der ipsilateralen Niere [2]. Die Symptome des Zinner-Syndroms reichen von perianalen und skrotalen Schmerzen über Hämatomspermie, Pollakisurie, Hämaturie zu Infertilität. In der Literatur sind etwa 200 Fälle als seltene Ursache der Verschlussazoospermie beschrieben. Das Zinner-Syndrom konnte in diesem Fall jedoch ausgeschlossen werden [1].

Weitere Ursachen der Samenblasenagenesie durch Entwicklungsstörungen der Embryonalzeit aufgrund exogener Umweltauslöser, welche zu Fehlbildungen des Wolff’schen Gangs und damit zu einer unilateralen Samenblasenagenesie führen, sind derweil nur wenig untersucht. Eine Studie bei 141 Patienten mit Infertilität zeigte bei 7 % der Patienten eine Samenblasenagenesie, die zu einem Großteil nur unilateral vorlag [3]. Auf eine Punktion des zystisch dilatieren linksseitigen Vas deferens zur Diagnostik auf vorhandene Spermien wurde aufgrund der möglicherweise rein diagnostischen und fraglich zielführenden therapeutischen Indikation auf Patientenwunsch in unserem Fall verzichtet.

Eine besondere diagnostische und therapeutische Herausforderung der Infertilität stellt sich insbesondere bei komplexen anatomischen Anomalien oder seltenen Ursachen. Umso wichtiger ist es hier, die gezielte Anamnese und eine weiterführende, auch bildgebende, Diagnostik im interdisziplinären Setting durchzuführen. Insbesondere genetische Alterationen sollten vor weiterer Therapieplanung evaluiert werden. Als gängige Methode der Spermiengewinnung bei Männern mit obstruktiver Azoospermie gilt die TESE. Je nach Studienlage variiert die Datenlage zur Erfolgsrate zwischen ca. 55 % bei einer regulären TESE und reicht bis zu 70–80 % bei einer Mikro-TESE [4, 5]. Bei Vorliegen einer Verschlussazoospermie zeigte sich in unserem Patientenbeispiel in der im Verlauf durchgeführten TESE eine regelrechte testikuläre Spermiogenese, so dass weitere Schritte im Rahmen der Kinderwunschbehandlung eingeleitet werden konnten.

## Fazit für die Praxis

- Dieser Fall verdeutlicht die Komplexität männlicher Infertilität und die Notwendigkeit einer umfassenden Diagnostik und Therapieplanung bei Patienten mit anatomischen Anomalien.
- Differentialdiagnosen einer Verschlussazoospermie, seltene Erkrankungen wie das Zinner-Syndrom oder das Vorliegen einer CFTR-Mutation („cystic fibrosis transmemrane conductance regulator“) sollten im Rahmen der Abklärung bedacht werden.
